# Crystal deposition triggers tubule dilation that accelerates cystogenesis in polycystic kidney disease

**DOI:** 10.1172/JCI193824

**Published:** 2025-05-01

**Authors:** Jacob A. Torres, Mina Rezaei, Caroline Broderick, Louis Lin, Xiaofang Wang, Bernd Hoppe, Benjamin D. Cowley, Vincenzo Savica, Vicente E. Torres, Saeed Khan, Ross P. Holmes, Michal Mrug, Thomas Weimbs

Original citation: *J Clin Invest*. 2019;129(10):4506-4522. https://doi.org/10.1172/JCI128503

Citation for this corrigendum: *J Clin Invest*. 2025;135(9):e193824. https://doi.org/10.1172/JCI193824

The dosing of a drug in the Methods and figure legends was incorrect. The corrected sentences are below.

A high dose of NaOx (70 mg/kg) was initially used to induce acute, severe nephrolithiasis in the mice, which led to rapid, abundant crystal deposition that appeared to be exclusively luminal (Figure 2, A and B).

WT male and female C57BL/6 mice (Charles River Laboratories), 8–10 weeks of age and weighing between 20 and 30 g, were challenged with a single i.p. injection of 0.22 M NaOx in 0.9% saline, sterile filtered and administered at 30 mg/kg (low dose), and euthanized at 6 hours (*n* = 4), 1 day (*n* = 12), 3 days (*n* = 8), and 7 days (*n* = 10); or at 70 mg/kg (high dose), and animals were euthanized at 3 hours (*n* = 4) and 1 day (*n* = 9) to induce acute CaOx crystal deposition.

Eight-week-old Sprague Dawley rats weighing between 200 and 300 g (Charles River Laboratories) were challenged with a single i.p. injection of 0.22 M NaOx in 0.9% saline, sterile filtered and administered at 70 mg/kg and were analyzed after 6 hours (*n* = 5), 1 day (*n* = 5), 3 days (*n* = 5), or 7 days (*n* = 3).

To induce moderate, reversible crystal deposition, we next administered a low dose of NaOx (30 mg/kg), which resulted in CaOx crystal deposition as early as 6 hours after injection, peaking at 24 hours, with excretion of most crystals by day 3 (Figure 2D).

Figure 2. (**A**) H&E-stained kidney sections 1 day after administration of 70 mg/kg NaOx, visualized by normal and polarized light microscopy.

Figure 2. (**C**) Immunoblot of total kidney lysates for p-S6 (Ser235/236), p-STAT3 (Tyr705), and total proteins 3 hours (*n* = 4) and 1 day (*n* = 9) after 70 mg/kg NaOx treatment.

Figure 2. (**D**) Polarized light micrographs of kidney sections from mice treated with 30 mg/kg NaOX, 6 hours (*n* = 4), 1 day (*n* = 12), 3 days (*n* = 8), and 7 days (*n* = 13) after treatment.

Figure 2. (**E**) Immunofluorescence staining of p-S6 (Ser235/236) and p-STAT3 (Tyr705) in mice treated with 30 mg/kg NaOx. Images of animals treated with 30 mg/kg NaOx are representative of 5 experiments.

Figure 3. (**A**) Polarized microscopic images of H&E-stained renal sections from rats treated with 70 mg/kg NaOx, 6 hours (*n* = 5), 24 hours (*n* = 5), 3 days (*n* = 5), and 7 days (*n* = 3) after treatment.

The authors regret the errors.

## Version 1 05/01/2025

Electronic publication

